# An open‐label, dose‐ranging study of Rolontis, a novel long‐acting myeloid growth factor, in breast cancer

**DOI:** 10.1002/cam4.1388

**Published:** 2018-03-23

**Authors:** Jeffrey L. Vacirca, Arlene Chan, Klára Mezei, Clarence S. Adoo, Zsuzsanna Pápai, Kimberly McGregor, Meena Okera, Zsolt Horváth, László Landherr, Jerzy Hanslik, Steven J. Hager, Emad N. Ibrahim, Makharadze Rostom, Gajanan Bhat, Mi Rim Choi, Guru Reddy, Karen L. Tedesco, Richy Agajanian, István Láng, Lee S. Schwartzberg

**Affiliations:** ^1^ New York Cancer Specialists East Setauket New York; ^2^ Breast Cancer Research Centre WA and Curtin University Perth Western Australia Australia; ^3^ Szabolcs‐Szatmár Bereg County Hospital and University Teaching Hospital Nyíregyháza Hungary; ^4^ Arizona Center for Cancer Care Glendale Arizona; ^5^ State Health Center Budapest Hungary; ^6^ Samaritan Hematology and Oncology Associates Corvalis Oregon; ^7^ Adelaide Cancer Centre Kurralta Park South Australia Australia; ^8^ University of Debrecen Debrecen Hungary; ^9^ Uzsoki Hospital Budapest Hungary; ^10^ Szpital Rejonowy Dzienny Oddzial Chemioterapii Raciborzu Poland; ^11^ California Cancer Associates for Research and Excellence Fresno California; ^12^ Emad Ibrahim MD, Inc. Redlands California; ^13^ Cancer Center of Adjara Autonomous Republic Batumi Georgia; ^14^ Spectrum Pharmaceuticals Irvine California; ^15^ New York Oncology Hematology (US Oncology/McKesson Specialty Health) Albany New York; ^16^ The Oncology Institute of Hope and Innovation Downey California; ^17^ National Institute of Oncology Budapest Hungary; ^18^ West Cancer Center Memphis Tennessee

**Keywords:** Breast cancer, eflapegrastim, neutropenia, Rolontis

## Abstract

This randomized, open‐label, active‐controlled study investigated the safety and efficacy of three doses of Rolontis (eflapegrastim), a novel, long‐acting myeloid growth factor, versus pegfilgrastim in breast cancer patients being treated with docetaxel and cyclophosphamide (TC). The primary efficacy endpoint was duration of severe neutropenia (DSN) during the first cycle of treatment. Patients who were candidates for adjuvant/neoadjuvant TC chemotherapy were eligible for participation. TC was administered on Day 1, followed by 45, 135, or 270 *μ*g/kg Rolontis or 6 mg pegfilgrastim on Day 2. Complete blood counts were monitored daily when the absolute neutrophil count (ANC) fell to <1.5 × 10^9^/L. Up to four cycles of TC were investigated. The difference in DSN (time from ANC <0.5 × 10^9^/L to ANC recovery ≥2.0 × 10^9^/L) between the Rolontis and pegfilgrastim groups was −0.28 days (confidence interval [CI]: −0.56, −0.06) at 270 *μ*g/kg, 0.14 days (CI: −0.28, 0.64) at 135 *μ*g/kg, and 0.72 days (CI: 0.19, 1.27) at 45 *μ*g/kg. Noninferiority to pegfilgrastim was demonstrated at 135 *μ*g/kg (*P = *0.002) and 270 *μ*g/kg (*P *<* *.001), with superiority demonstrated at 270 *μ*g/kg (0.03 days; *P = *0.023). The most common treatment‐related adverse events (AEs) were bone pain, myalgia, arthralgia, back pain, and elevated white blood cell counts, with similar incidences across groups. All doses of Rolontis were well tolerated, and no new or significant treatment‐related toxicities were observed. In Cycle 1, Rolontis demonstrated noninferiority at the 135 *μ*g/kg dose and statistical superiority in DSN at the 270 *μ*g/kg dose when compared to pegfilgrastim.

## Introduction

Many effective chemotherapy regimens induce myelosuppressive side effects that can result in treatment delays, dose reductions, and an overall inability to complete treatment, which may compromise clinical outcomes. Myeloid growth factors, a class of biologic agents that regulate the proliferation, differentiation, survival, and activation of cells in the myeloid lineage, are often administered prophylactically to reduce the incidence of severe neutropenia, one of the most common and dose‐limiting myelosuppressive side effects of anticancer chemotherapy 1. Chemotherapy‐induced neutropenia can progress to febrile neutropenia, defined as a single temperature >38.3°C (or ≥38.0°C for >1 h) that presents with concurrent grade 3/4 neutropenia (absolute neutrophil count [ANC] ≤1.0 × 10^9^/L) 2, 3. As patients who develop febrile neutropenia are at risk for hospitalization, additional morbidities and even mortality, mitigation of the severity, and duration of this condition have been shown to improve chemotherapy treatment compliance and, ultimately, patient survival 4, 5.

Several growth factors are currently approved or are under clinical development in the United States (US) for the management of chemotherapy‐induced neutropenia. Filgrastim (Neupogen®, Amgen, Thousand Oaks, CA) is the methionylated recombinant human granulocyte‐colony stimulating factor (G‐CSF) expressed in *E. coli* and is administered as a daily subcutaneous injection (or by intravenous infusion) in conjunction with myelosuppressive chemotherapy to increase the circulating levels of neutrophils and thereby reduce the duration of severe neutropenia 6. However, a full response to filgrastim requires multiple daily injections due to a short (several hours) half‐life. Pegfilgrastim (Neulasta®, Amgen, Thousand Oaks, CA), the polyethylene glycol‐conjugated form of G‐CSF, is a long‐acting myeloid growth factor that is administered subcutaneously (SC) on a more convenient, less frequent dosing schedule (i.e., as a single dose administered once‐per‐cycle of chemotherapy) and has demonstrated improved reduction of neutropenia over both placebo and filgrastim 7, 8, 9, 10, 11, 12, 13. Two additional (currently not approved in the US) glyco‐pegylated growth factors, lipegfilgrastim and balugrastim, as well as a pegfilgrastim biosimilar, LA‐EP2006, have demonstrated noninferiority to pegfilgrastim as once‐per‐cycle administrations in Phase 3 trials conducted in patients with breast cancer 14, 15.

Rolontis™ (Spectrum Pharmaceuticals, Inc., Henderson, NV) (eflapegrastim, SPI‐2012, HM10460A) was developed by conjugating the recombinant human G‐CSF analog (17th_,_ 65th Ser‐G‐CSF, no additional N‐terminal Met) and the human immunoglobulin G4 Fc fragment via a 3.4 kDa Peg linker to produce a longer‐acting G‐CSF. In preclinical studies, Rolontis has demonstrated similar in vitro activity and a similar pharmacokinetic (PK) profile compared with pegfilgrastim 16. The in vivo and clinical potency of Rolontis, however, have been observed to be significantly higher than pegfilgrastim. Pharmacokinetic analysis of Rolontis in vivo revealed a twofold to threefold increase in area under the time‐concentration curve (AUC) for ANC compared to pegfilgrastim when administered at similar doses (when comparing the concentration of the G‐CSF molecule) in neutropenic mice and rats, normal rats, and monkeys. Furthermore, the duration of severe neutropenia in neutropenic mice and rats was significantly reduced compared to either pegfilgrastim or filgrastim 17, 18.

A randomized, double‐blind, placebo‐controlled, escalating single‐dose study with Rolontis was conducted in 40 healthy Korean subjects. Doses of Rolontis ranged from 5 to 350 *μ*g/kg. Rolontis showed dose‐dependent pharmacokinetic properties, and the area under the effect‐time curve (AUEC_last_) of both the absolute neutrophil count (ANC) and the CD34^+^ cell count increased as the dose increased 12.

The current Phase 2 study was designed to evaluate the efficacy and safety of Rolontis at three dose levels, as compared with pegfilgrastim, in patients with breast cancer receiving myelosuppressive docetaxel and cyclophosphamide (TC) chemotherapy. The clinical development of Rolontis is based upon the potential viability of Rolontis as a more potent, long‐acting alternative to pegfilgrastim that provides clinical benefits at a lower G‐CSF dose.

## Materials and Methods

### Patient population

This was a randomized, open‐label, multicenter, dose‐ranging, active‐controlled study of Rolontis versus pegfilgrastim in patients aged ≥18 years with breast cancer who were candidates for adjuvant or neoadjuvant treatment with TC chemotherapy (NCT01724866). Other key inclusion criteria included the willingness and ability to provide written informed consent; Eastern Cooperative Oncology Group (ECOG) performance status ≤2; adequate bone marrow function before the start of chemotherapy (ANC ≥1.5 × 10^9^/L, platelet count ≥100 × 10^9^/L, hemoglobin >9 g/dL); creatinine ≤1.5× upper limit of normal (ULN); total bilirubin ≤1.5 mg/dL; aspartate aminotransferase (AST) and/or alanine aminotransferase (ALT) ≤2.5 × ULN; and alkaline phosphatase ≤1.5 × ULN. Key exclusion criteria included a known sensitivity to *E coli*‐derived products, L‐asparanginase, somatropin growth hormone, or recombinant interferon *α*‐2b; an active infection or positive serology for human immunodeficiency virus, hepatitis B or hepatitis C; prior bone marrow or stem cell transplant; major surgery (except for breast surgery related to the patient's breast cancer diagnosis) within 4 weeks prior to enrollment; any other malignancy within 5 years prior to enrollment; pregnancy or breastfeeding; prolonged exposure to glucocorticosteroids and immunosuppressive agents; or dementia or significantly altered mental status that would prohibit the understanding and giving of informed consent or limit study compliance.

The study protocol and patient materials were approved by institutional review boards (IRBs) and/or ethics committees at all sites. The study conducted followed International Conference on Harmonization (ICH) Guidelines for Good Clinical Practice, including written informed consent and monitoring of all data.

### Treatment

Following a screening period of 30 days, eligible patients were sequentially assigned to receive SC injections of one of three weight‐based doses of Rolontis (45, 135, or 270 *μ*g/kg) or a fixed dose of 6 mg of pegfilgrastim. On Day 1 of each cycle, patients were treated with docetaxel (75 mg/m^2^) and cyclophosphamide (600 mg/m^2^) by IV infusion every 3 weeks for up to four cycles. On Day 2 of each cycle (approximately 24 h after TC chemotherapy), patients administered their assigned study treatment with either Rolontis or pegfilgrastim.

### Methods of assessment

Patients were monitored for adverse events (AEs) continuously for the duration of the study. In addition, clinical laboratory measurements were performed during every cycle, including a complete blood count (CBC) with differential, serum chemistry, and urinalysis. Adverse events and laboratory values were graded according to the National Cancer Institute (NCI) Common Terminology Criteria for Adverse Events (CTCAE), version 4.03 2.

Efficacy (i.e., duration of severe neutropenia) was evaluated through CBCs with differential performed pretreatment and on Days 1, 2, and 3 of every cycle. For patients with an ANC ≥1.5 × 10^9^/L on Day 3, subsequent CBCs were performed twice weekly. If at any time ANC was <1.5 × 10^9^/L, daily CBCs were performed until ANC recovered to ≥1.5 × 10^9^/L.

### Study endpoints

The primary efficacy endpoint was the duration of severe neutropenia (DSN) in Cycle 1, with severe neutropenia defined as ANC <0.5 × 10^9^/L (grade 4 per NCI CTCAE) and DSN defined as the interval from the day of first observation of grade 4 neutropenia to first ANC recovery to ≥2.0 × 10^9^/L.

Secondary endpoints assessed for each cycle included the following: (1) time to ANC recovery, defined as the time from chemotherapy administration until an increase in ANC to ≥2.0 × 10^9^/L after the expected nadir (date of ANC recovery – date of chemotherapy +1); (2) depth of ANC nadir, defined as the lowest ANC value; (3) time to ANC nadir, defined as the time from chemotherapy administration until the occurrence of the ANC nadir (date of ANC nadir – date of chemotherapy +1); (4) incidence of febrile neutropenia, defined as a temperature of >38.2°C occurring within 1 day of an ANC <0.5 × 10^9^/L, and/or a reported AE of febrile neutropenia; and (5) hospitalizations for any reason, including febrile neutropenia.

Blood samples for immunogenicity analysis were collected before the start of each chemotherapy cycle (Day 1) and at the end‐of‐study visit.

### Statistical methods

Sample size estimates were based on a noninferiority design. It was assumed that the pooled standard deviation of DSN for Cycle 1 would be 2.1 days, estimated from previous pegfilgrastim studies 19. The total planned sample size of 144 patients provided an 80% power to establish noninferiority, based on the one‐sided 95% upper confidence limit of the difference in the mean DSN of pooled experimental arms and mean DSN of the control arm being <1 day.

Treatment differences in DSN in Cycle 1 were analyzed using confidence intervals (CIs) calculated based upon 10,000 bootstrap samples stratified by baseline weight (<65 kg, ≥65 kg and ≤75 kg, or >75 kg). Two‐sided 95% CIs for the difference in mean DSN between each Rolontis group and the pegfilgrastim group were calculated. Noninferiority was demonstrated if the upper limit of the two‐sided 95% CI was <1 day. Two types of *P*‐values were reported: The noninferiority *P*‐value was calculated as two times the proportion of treatment difference >1 in the resampling, and the superiority *P*‐value was calculated as two times the proportion of treatment difference >0 in the resampling.

Descriptive statistics (*n*, mean, median, and standard deviation) were used for all continuous variables.

## Results

### Patient population

**Figure 1:**
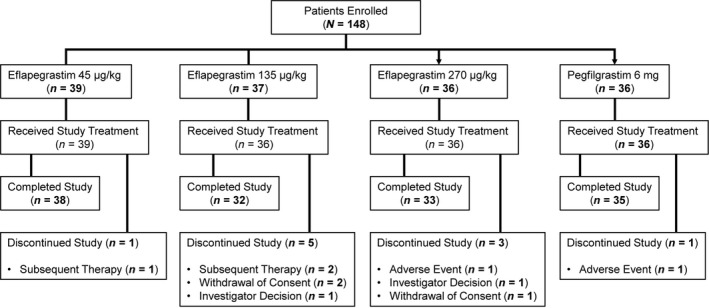
Patient disposition. CONSORT (Consolidated Standards of Reporting Trials) trial flow diagram.

The study enrolled 148 patients between March 2013 and August 2014 at 27 study sites in six countries (Australia, Georgia, Hungary, Israel, Poland, and the USA); all enrolled patients were included in the safety population (Fig. 1). The evaluable population comprised 147 patients who received at least one treatment with TC chemotherapy and at least one dose of either pegfilgrastim (*n* = 36) or Rolontis (*n* = 39 at 45 *μ*g/kg, *n* = 36 at 135 *μ*g/kg and *n* = 36 at 270 *μ*g/kg) (Table 1).

**Table 1:** Demographics and other baseline characteristics (evaluable population)

	Rolontis dose			Pegfilgrastim 6 mg (*n* = 36)	Total (*N* = 147)
	45 *μ*g/kg (*n* = 39)	135 *μ*g/kg (*n* = 36)	270 *μ*g/kg (*n* = 36)		
Age (years)					
Median (range)	62 (33–77)	59 (32–74)	57 (38–77)	61 (35–77)	59 (32–77)
Gender, *n* (%)					
Female	39 (100)	35 (97)	34 (94)	36 (100)	144 (98)
Male	0	1 (3)	2 (6)	0	3 (2)
Race, *n* (%)					
White	36 (92)	36 (100)	35 (97)	32 (89)	139 (95)
Black or African‐American	2 (5)	0	0	0	2 (1)
Other	1 (3)	0	1 (3)	4 (11)0	6 (4)
ECOG PS, *n* (%)					
0 (fully active)	33 (85)	32 (89)	35 (97)	33 (92)	133 (90)
1 (restricted)	5 (13)	4 (11)	1 (3)	2 (6)	12 (8)
2 (ambulatory)	1 (3)	0	0	0	1 (1)
Missing	0	0	0	1 (3)	1 (1)
Disease Stage, *n* (%)					
I	8 (21)	7 (19)	6 (17)	9 (25)	30 (20)
IIA	12 (31)	12 (33)	13 (36)	10 (28)	47 (32)
IIB	8 (21)	11 (31)	11 (31)	7 (19)	37 (25)
IIIA	6 (15)	3 (8)	2 (6)	6 (17)	17 (12)
IIIB	3 (8)	3 (8)	2 (6)	1 (3)	9 (6)
IIIC	2 (5)	0	2 (6)	1 (3)	5 (3)
IV	0	0	0	2 (6)	2 (1)
WHO Classification, *n* (%)					
Invasive ductal carcinoma	31 (79)	27 (75)	32 (89)	33 (92)	123 (84)
Invasive lobular carcinoma	3 (8)	3 (8)	0	2 (6)	8 (5)
Carcinoma with metaplasia	1 (3)	0	0	0	1 (1)
Medullary carcinoma	0	1 (3)	0	0	1 (1)
Other	4 (10)	5 (14)	4 (11)	1 (3)	14 (10)
HER2 Status, *n* (%)					
HER2+	1 (3)	7 (19)	6 (17)	3 (8)	17 (12)
HER2−	9 (23)	9 (25)	9 (25)	10 (28)	37 (25)
Unknown	29 (74)	20 (56)	21 (58)	23 (64)	94 (64)

ECOG, Eastern Cooperative Oncology Group; PS, performance status; SD, standard deviation; WHO, World Health Organization.

Demographics and baseline characteristics were generally well balanced across treatment groups, as shown in Table 1. Patient ages ranged from 32 to 77 years, with a median of 59 years. Most patients were female (98%) and White (95%), and the majority (84%) had invasive ductal carcinoma. Disease stage at the time of breast cancer diagnosis was Stage II (57%), Stage I (20%), or Stage III (21%), with two patients (1%) diagnosed as Stage IV. Hormone receptor status was positive for 55% of patients (estrogen receptor +/progesterone receptor +), 17% of patients had ER+/PR‐ disease, and 28% of patients had ER‐/PR‐ disease or unknown hormone receptor status. Human epidermal growth factor receptor 2 (HER2) status was positive for 12% of patients, negative for 25% of patients, and unknown for 63% of patients. Most patients (90% overall) had a baseline Eastern Cooperative Oncology Group (ECOG) status of 0, 12 patients (8%) had a performance status of 1, and one patient (1%) had a performance status of 1.

### Chemotherapy relative dose intensity

In Cycle 1, only one patient (3%), in the 135 *μ*g/kg Rolontis group, discontinued from the study after receiving TC therapy but before receiving Rolontis treatment. Overall, 138 patients (93%) completed all four cycles of TC chemotherapy, with 10 patients (7%) discontinuing treatment prematurely. Treatment delivery of docetaxel and cyclophosphamide was >97% over all four cycles, with nine patients (6%) experiencing TC dose interruptions and five patients (3%) requiring TC dose delays. The median dosing compliance with Rolontis and pegfilgrastim was 100% across all four cycles, with dose delays due to nontreatment‐related AEs required for one patient in the 45 *μ*g/kg Rolontis group and two patients in the 270 *μ*g/kg Rolontis group.

### Efficacy

#### Duration of severe neutropenia

In the 270 *μ*g/kg Rolontis group, severe neutropenia was reported in one patient (3%) and lasted 1 day (Table 2). In the lowest Rolontis dose group (45 *μ*g/kg Rolontis), approximately one‐third of patients in (14 patients, 36%) experienced severe neutropenia that lasted between 1 and 5 days. In the 135 *μ*g/kg Rolontis group, seven patients (19%) experienced severe neutropenia, which lasted for 1 to 2 days in six patients (17%) and 7 days in one patient, 3%). In the pegfilgrastim group, five patients (14%) experienced severe neutropenia that lasted for 1–3 days.

**Table 2:** Duration of severe neutropenia (evaluable population)

DSN (days)	Rolontis dose			Pegfilgrastim 6 mg (*n* = 36)
	45 *μ*g/kg (*n* = 39)	135 *μ*g/kg (*n* = 36)	270 *μ*g/kg (*n* = 36)	
Cycle 1				
0 days, *n* (%)	25 (64)	29 (81)	35 (97)	31 (86)
1 day, *n* (%)	1 (3)	3 (8)	1 (3)	1 (3)
2 days, *n* (%)	5 (13)	3 (8)	0	2 (6)
3 days, *n* (%)	5 (13)	0	0	2 (6)
≥4 days, *n* (%)	3 (8)*	1 (3)†	0	0
*n*	39	36	36	36
Mean ± SD (days)	1.03 ± 1.55	0.44 ± 1.28	0.03 ± 0.17	0.31 ± 0.82
Difference with pegfilgrastim	0.72	0.14	−0.28	
95% CI	(0.19, 1.27)	(−0.28, 0.64)	(−0.56, −0.06)	
Noninferiority *P*‐value	0.296	0.002	<0.001	
Superiority *P*‐value	0.006	0.528	0.023	
Cycle 2				
*n*	39	34	34	36
Mean ± SD (days)	0.46 ± 1.02	0.12 ± 0.48	0.03 ± 0.17	0.08 ± 0.37
Difference with pegfilgrastim	0.38	0.04	−0.05	
95% CI	(0.06, 0.74)	(−0.16, 0.24)	(−0.19, 0.06)	
Noninferiority *P*‐value	0.001	<0.001	<0.001	
Superiority *P*‐value	0.019	0.649	0.563	
Cycle 3				
*n*	38	32	34	36
Mean ± SD (days)	0.45 ± 1.13	0.16 ± 0.63	0.15 ± 0.61	0.14 ± 0.59
Difference with pegfilgrastim	0.31	0.02	0.01	
95% CI	(−0.07, 0.72)	(−0.27, 0.30)	(−0.27, 0.28)	
Noninferiority *P*‐value	0.002	<0.001	<0.001	
Superiority *P*‐value	0.126	0.882	0.899	
Cycle 4				
*n*	38	32	33	35
Mean ± SD (days)	1.05 ± 4.58	0.19 ± 0.74	0.09 ± 0.52	0.11 ± 0.40
Difference with pegfilgrastim	0.94	0.07	−0.02	
95% CI	(−0.01, 2.47)	(−0.17, 0.38)	(−0.23, 0.22)	
Noninferiority *P*‐value	0.781	<0.001	<0.001	
Superiority *P*‐value	0.061	0.605	0.848	

CI, confidence interval; DSN, duration of severe neutropenia; SD, standard deviation. Median DSN was 0 for all treatment groups in all cycles.

*4 days (*n* = 1); 5 days (*n* = 2); ^†^7 days (*n* = 1).

A dose‐effect trend in the mean DSN in Cycle 1 was observed across the three doses of Rolontis with the DSN decreasing with increasing dose: 1.03 days at 45 *μ*g/kg, 0.44 days at 135 *μ*g/kg, and 0.03 days at 270 *μ*g/kg. The difference in Cycle 1 DSN between the Rolontis groups and the pegfilgrastim group was −0.28 days (CI: −0.56, −0.06) at 270 *μ*g/kg of Rolontis, 0.14 days (CI: −0.28, 0.64) at 135 *μ*g/kg of Rolontis, and 0.72 days (CI: 0.19, 1.27) at 45 *μ*g/kg of Rolontis (Table 2). The upper limit of the two‐sided 95% CI for the difference was >1 day for the 45 *μ*g/kg group, but <1 day for the 135 *μ*g/kg and 270 *μ*g/kg groups. Therefore, in Cycle 1, noninferiority to pegfilgrastim was demonstrated for the 135 *μ*g/kg (*P = *0.002) and 270 *μ*g/kg (*P *<* *0.001) Rolontis groups, but not for the 45 *μ*g/kg Rolontis group (*P *= 0.296). Superiority was demonstrated in the 270 *μ*g/kg Rolontis group (0.03 days) compared to the pegfilgrastim group (0.31 days; *P *=* *0.023).

During Cycles 2 and 3, all three Rolontis dose groups met the criterion for noninferiority to the pegfilgrastim group (Table 2), as the upper limit of the 95% CI for the differences from the pegfilgrastim group was <1 day. During Cycle 4, the noninferiority criterion was met in the 135 *μ*g/kg (*P *<* *0.001) and 270 *μ*g/kg (*P *<* *0.001) Rolontis groups, but not the 45 *μ*g/kg Rolontis group (*P = *0.781).

#### Absolute neutrophil count

**Figure 2:**
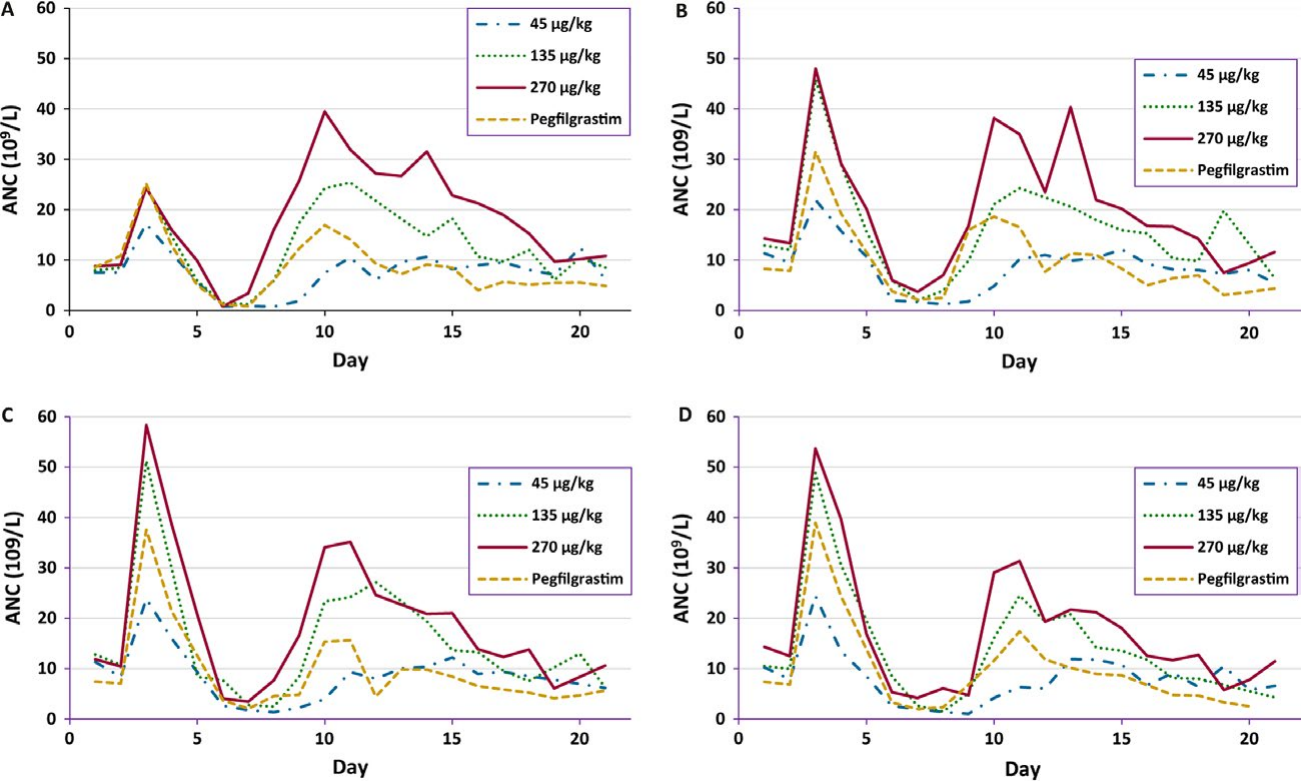
Median absolute neutrophil count by treatment over time (evaluable population). Absolute neutrophil count in patients treated with 45, 135, and 270 *μ*g/kg Rolontis or pegfilgrastim by treatment group in (A) Cycle 1, (B) Cycle 2, (C) Cycle 3, and (D) Cycle 4.

The changes in median ANC over time were biphasic and were similar in all four treatment groups during all four cycles. In Cycle 1, the ANC nadir occurred at approximately Day 6 to Day 9, with two separate peaks with the first peak observed on Day 3 and the second peak observed between Days 10 and 13 (Fig. 2A). In all treatment groups, the ANC peak on Day 3 increased in each cycle and reached a maximum by Cycle 3. The postnadir ANC recovery peak was approximately the same level in each cycle. The ANC values in patients receiving the two higher doses of Rolontis, 135 and 270 *μ*g/kg, were higher than the values in patients receiving pegfilgrastim on most days during each cycle. Patients receiving 45 *μ*g/kg of Rolontis showed slightly lower ANC values than patients on pegfilgrastim during the first half of each cycle but the values were similar in the second half of each cycle.

For patients whose ANC decreased below 2.0 × 10^9^/L in Cycle 1, the median time to ANC recovery in the 45 *μ*g/kg Rolontis group (Day 10) was significantly longer than in the pegfilgrastim arm (Day 9; *P *=* *0.002; hazard ratio [HR] 1.4) (Table 3). No significant differences from the pegfilgrastim group in median time to ANC recovery in Cycle 1 were observed in the 135 *μ*g/kg Rolontis group (Day 8.5; *P = *0.711; HR 0.9), and the median time to ANC recovery was significantly shorter in the 270 *μ*g/kg Rolontis group (Day 8; *P *=* *0.028; HR 0.3) compared to the pegfilgrastim group. In Cycles 2 through 4, only the median time to ANC recovery in the 45 *μ*g/kg Rolontis group (Day 11, Cycle 4) was significantly different from the pegfilgrastim group (Day 10; *P = *0.009; HR 1.4).

**Table 3:** Absolute neutrophil count in cycle 1 (evaluable population)

DSN (days)	Rolontis dose			Pegfilgrastim 6 mg (*n* = 36)
	45 *μ*g/kg (*n* = 39)	135 *μ*g/kg (*n* = 36)	270 *μ*g/kg (*n* = 36)	
Cycle 1				
Depth of ANC Nadir				
*n*	39	36	36	36
Median Depth (× 10^9^/L)	0.8	3.0	6.2	3.0
Min, Max (× 10^9^/L)	0.0, 9.0	0.1, 14.1	0.2, 21.0	0.0, 9.1
Ratio to Pegfilgrastim (95% CI)	0.4	1.0	2.5	–
*P*‐value Time to ANC Recovery (Days)	0.008	0.911	0.002	–
Time to ANC Nadir				
Median (95%CI) (× 10^9^/L)	8.0 (7–8)	7.0 (nc)	7.0 (7–8)	7.5 (7–16)
Time to ANC Recovery (Days)				
*n*	29	14	6	14
Median Time to ANC Recovery (95% CI)	10.0 (10–11)	8.5 (8–9)	8.0 (7–9)	9.0 (8–10)
HR to Pegfilgrastim (95% CI)	1.4 (1.1–1.8)	0.9 (0.6–1.4)	0.3 (0.1–0.9)	**–**
*P*‐value	0.002	0.711	0.028	**–**

CI, confidence interval; DSN, duration of severe neutropenia; SD, standard deviation.

During Cycle 1, the median depth of the ANC nadir was 6.2 × 10^9^/L for the 270 *μ*g/kg Rolontis group, 3.0 × 10^9^/L for the 135 *μ*g/kg Rolontis group, 0.8 × 10^9^/L for the 45 *μ*g/kg Rolontis group, and 3.0 × 10^9^/L for the pegfilgrastim group. The depth of ANC nadir in the 270 *μ*g/kg Rolontis group was significantly higher than in the pegfilgrastim arm in Cycle 1 (*P *=* *0.002), Cycle 2 (*P *=* *0.027), and Cycle 4 (*P *=* *0.005). The median time to ANC nadir in Cycle 1 was 7 days for the 135 and 270 *μ*g/kg Rolontis groups, 8 days for the 45 *μ*g/kg Rolontis group, and 7.5 days for the pegfilgrastim group, with similar ANC nadir depth and time results patterns observed during Cycles 2 through 4.

#### Febrile neutropenia

The incidence of febrile neutropenia was low in all treatment groups across all cycles and was reported in two patients (6%) in the pegfilgrastim group compared with one patient (3%) each in the Rolontis 135 and 270 *μ*g/kg groups and three patients (8%) in the 45 *μ*g/kg group. The incidents of FN in the 135 and 270 *μ*g/kg Rolontis groups and in two of the patients in the 45 *μ*g/kg Rolontis group occurred in Cycle 1. One patient in the 45 *μ*g/kg Rolontis group experienced FN in both Cycle 2 and Cycle 3, and one of the patients in the pegfilgrastim group experienced FN in Cycle 3 and the other in Cycle 4. The overall rate of hospitalizations across all cycles (8%) was also similar across treatment groups, with the highest incidence observed in the pegfilgrastim group (five patients, 14%) and the lowest incidence observed in the Rolontis 270 *μ*g/kg group (one patient, 3%). Three patients in the 45 *μ*g/kg Rolontis group were hospitalized, but only one patient was hospitalized due to febrile neutropenia. No statistically significant differences in either febrile neutropenia incidence or hospitalizations were observed between any Rolontis dose level and pegfilgrastim.

### Adverse events

Most patients experienced treatment‐emergent adverse events (TEAEs) and the incidence of TEAEs was similar between treatment groups (Table 4). More Grade 3–4 TEAEs were reported in the 45 *μ*g/kg Rolontis group than in the other treatment groups. The most commonly reported TEAEs were fatigue, alopecia, nausea, diarrhea, and bone pain. Treatment‐related TEAEs were reported in >50% of patients in all treatment groups. The most frequently reported treatment‐related TEAE in all four treatment groups was bone pain, which was reported in more than 20% of patients in all four treatment groups (Table 5). No patients discontinued from the study due to treatment‐related AEs. No clinically meaningful differences in TEAEs (incidence or type) were observed overall or for individual events across Rolontis dose levels or between treatment types. Serious adverse events (SAEs) were reported for 13% of patients across cohorts, with treatment‐related SAEs observed only in the pegfilgrastim group (Table 4). Few patients required treatment discontinuation for AEs, none of which were considered treatment‐related, and no disease progression patient deaths or events were reported.

**Table 4:** Overview of treatment‐emergent adverse events (safety population)

Preferred term	Rolontis dose			Pegfilgrastim 6 mg (*n* = 36) *n* (%)
	45 *μ*g/kg (*n* = 39) *n* (%)	135 *μ*g/kg (*n* = 37) *n* (%)	270 *μ*g/kg (*n* = 36) *n* (%)	
Any treatment‐emergent adverse event	36 (92)	33 (89)	33 (92)	35 (97)
Grade 3–4 TEAE	24 (62)	12 (32)	13 (36)	12 (33)
Treatment‐related TEAE	20 (51)	19 (51)	23 (64)	21 (58)
Any Grade 3–4 related TEAE	4 (10)	3 (8)	4 (11)	1 (3)
Discontinuation due to TEAE	0	2 (5)	1 (3)	1 (3)
Any Serious Adverse Event SAE	5 (13)	4 (11)	2 (6)	8 (22)
Any Treatment‐related SAE	0	0	0	3 (8)

**Table 5:** Treatment‐related, treatment‐emergent adverse events with incidence ≥10% (safety population)

Preferred term	Rolontis dose			Pegfilgrastim 6 mg (*n* = 36) *n* (%)
	45 *μ*g/kg (*n* = 39) *n* (%)	135 *μ*g/kg (*n* = 37) *n* (%)	270 *μ*g/kg (*n* = 36) *n* (%)	
Any treatment‐related treatment‐emergent adverse event	20 (51)	19 (51)	23 (64)	21 (58)
Bone pain	8 (21)	8 (22)	9 (25)	10 (28)
Arthralgia	5 (13)	5 (14)	3 (8)	5 (14)
Myalgia	5 (13)	1 (3)	4 (11)	7 (19)
Back pain	6 (15)	4 (11)	1 (3)	3 (8)
Leukocytosis	2 (5)	3 (8)	7 (19)	2 (6)
Headache	4 (10)	2 (5)	3 (8)	4 (11)
Fatigue	6 (15)	2 (5)	0	1 (3)
Pyrexia	0	1 (3)	2 (6)	4 (11)

### Immunogenicity

Serum samples from 143 patients were tested for antidrug antibodies (ADA) to Rolontis and G‐CSF. No neutralizing antibodies against Rolontis or G‐CSF were detected in patients administered Rolontis in this study.

## Discussion

Prophylactic administration of myeloid growth factors has become standard treatment to reduce the incidence of chemotherapy‐induced severe neutropenia, particularly recommended for therapies associated with a ≥20% risk of febrile neutropenia due to both chemotherapy regimen and individual patient risk factors 1, 3, 20. Rolontis is being developed as a novel long‐acting form of G‐CSF that has a distinct structure compared to pegfilgrastim and may represent a more potent new G‐CSF that delivers improved clinical efficacy.

The primary objective of the current open‐label, multicenter, dose‐ranging, active‐controlled study was to evaluate three different dose levels of Rolontis on the DSN (time from ANC <0.5 × 10^9^/L to ANC ≥2.0 × 10^9^/L) during Cycle 1 of treatment in patients with breast cancer who were receiving adjuvant or neoadjuvant TC chemotherapy. Several Phase 3 randomized, blinded studies conducted with other novel long‐acting myeloid growth factors or a pegfilgrastim biosimilar investigated the same endpoint in patients with breast cancer receiving myelosuppressive chemotherapy. These studies suggested other long‐acting growth factors could have similar effects on the duration of DSN in Cycle 1 of TC therapy 14, 15, 21.

The mean Cycle 1 DSNs observed with Rolontis in the current study were comparable or lower than what was demonstrated for pegfilgrastim. At the two higher doses of Rolontis studied, the mean Cycle 1 DSNs were statistically noninferior to pegfilgrastim, and the Cycle 1 DSN with the 270 *μ*g/kg Rolontis dose was statistically superior to pegfilgrastim (*P = *0.023). In Cycles 2, 3, and 4, the DSN with 135 *μ*g/kg Rolontis and 270 *μ*g/kg Rolontis were noninferior to pegfilgrastim and the DSN with 45 *μ*g/kg Rolontis was noninferior to pegfilgrastim in Cycles 2 and 3.

In Cycle 1, the majority of the evaluable population, both overall and in each group, did not experience any severe neutropenia, with the highest proportion of patients experiencing severe neutropenia in the Rolontis 45 *μ*g/kg group (36%) along with the 135 *μ*g/kg (19%) and 270 *μ*g/kg (3%). Rolontis groups and 14% of the pegfilgrastim group indicate a dose proportional reduction of severe neutropenia by Rolontis. This was due in part to the study design which did not mandate daily CBC collections until the ANC <1.5 × 10^9^/L. In subsequent cycles, the DSN was generally shorter across all arms, consistent with other studies using the same myelosuppressive chemotherapy 7, 22. The incidences of febrile neutropenia and overall hospitalizations were low (5% and 8%, respectively, in the overall evaluable population).

All doses of Rolontis and pegfilgrastim were well tolerated. While most of the patients in all four groups (≥89%) experienced TEAEs, approximately one‐half of patients (51% to 64%) experienced AEs that were considered to be treatment‐related. The most frequently observed TEAEs (in ≥20% of patients) in all four treatment groups included fatigue, alopecia, nausea, diarrhea, and bone pain. Most of these events (fatigue, alopecia, nausea, and diarrhea) are typically associated with TC treatment. Bone pain is an expected event associated with myeloid growth factors due to the mechanism of action of this class of agents 3. Twenty‐one to 25% of patients receiving Rolontis exhibited bone pain compared to 28% of patients receiving pegfilgrastim.

Adverse events were primarily mild to moderate in severity, with few patients experiencing SAEs or TEAEs leading to drug modification or discontinuation across all treatment groups. No trends in TEAE incidences were observed overall or for most individual events, although insomnia and pyrexia were reported more frequently in the pegfilgrastim (31% and 22%, respectively) than Rolontis groups (pooled 12% and 9%, respectively). Neutropenia and decreased neutrophil count were more frequently reported in the 45 *μ*g/kg Rolontis group (28% and 33%, respectively) than any other group, likely due to suboptimal dose level, which is consistent with the longer DSN, lower ANC levels, and deeper ANC nadir observed in this low Rolontis dose group. No neutralizing antibodies against Rolontis or G‐CSF were detected in patients administered Rolontis in this study.

Rolontis is being further investigated in two currently ongoing randomized, Phase 3 trials. The results from this study show that the dose range of 135–270 *μ*g/kg was both efficacious and safe. Based on these results, a dose of 13.2 mg/0.6 mL/dose, equivalent to 3.6 mg G‐CSF, was selected for the Phase 3 development program. This dose is equivalent to approximately 176 *μ*g/kg for a 75 kg patient and is being administered in two ongoing Phase 3 studies with Rolontis.

The results from this study indicate that Rolontis, a novel long‐acting G‐CSF, conferred comparable efficacy benefits to pegfilgrastim on duration of severe neutropenia and presented no new safety concerns when administered to patients with breast cancer receiving docetaxel and cyclophosphamide as adjuvant or neoadjuvant chemotherapy.

## Conflict of Interest

Drs Vacirca, Chan, Mezei, Adoo, Papai, McGregor, Okera, Horvath, Landherr, Hanslik, Hager, Ibrahim, Rostom, Tedesco, Agajanian, Lang, and Schwartzberg have disclosed no conflicts of interest. Drs. Bhat, Choi, and Reddy are employees of Spectrum Pharmaceuticals, Inc.
